# Glycerol induced paraspinal muscle degeneration leads to hyper-kyphotic spinal deformity in wild-type mice

**DOI:** 10.1038/s41598-023-35506-9

**Published:** 2023-05-20

**Authors:** Alex M. Noonan, Emily Buliung, K. Josh Briar, Diana Quinonez, Cheryle A. Séguin, Stephen H. M. Brown

**Affiliations:** 1 Department of Human Health and Nutritional Sciences, University of Guelph, Guelph, ON Canada; 2 Department of Physiology and Pharmacology, Schulich School of Medicine and Dentistry, Bone and Joint Institute, University of Western Ontario, London, ON Canada

**Keywords:** Musculoskeletal system, Physiology

## Abstract

Degenerative spinal disorders, including kyphotic deformity, are associated with a range of degenerative characteristics of the paraspinal musculature. It has therefore been hypothesized that paraspinal muscular dysfunction is a causative factor for degenerative spinal deformity; however, experimental studies demonstrating causative relationships are lacking. Male and female mice received either glycerol or saline injections bilaterally along the length of the paraspinal muscles at four timepoints, each separated by 2 weeks. Immediately after sacrifice, micro-CT was performed to measure spinal deformity; paraspinal muscle biopsies were taken to measure active, passive and structural properties; and lumbar spines were fixed for analysis of intervertebral disc (IVD) degeneration. Glycerol-injected mice demonstrated clear signs of paraspinal muscle degeneration and dysfunction: significantly (*p* < 0.01) greater collagen content, lower density, lower absolute active force, greater passive stiffness compared to saline-injected mice. Further, glycerol-injected mice exhibited spinal deformity: significantly (*p* < 0.01) greater kyphotic angle than saline-injected mice. Glycerol-injected mice also demonstrated a significantly (*p* < 0.01) greater IVD degenerative score (although mild) at the upper-most lumbar level compared to saline-injected mice. These findings provide direct evidence that combined morphological (fibrosis) and functional (actively weaker and passively stiffer) alterations to the paraspinal muscles can lead to negative changes and deformity within the thoracolumbar spine.

## Introduction

With a growing aged population, the number of patients being treated for degenerative spinal disorders is expected to increase^1^, thus creating a significant burden on quality of life and social costs^2,3^. Degenerative spinal disorders are a multifactorial condition that are associated with a spectrum of indications related to skeletal muscle, including greater paraspinal fat^4,5^ and collagen content^6,7^, lower paraspinal muscle strength^8^, and altered paraspinal passive mechanical properties^9^. This isn’t surprising, as many chronic diseases are associated with skeletal muscle alterations^10–13^; degenerative spinal disorders are no exception. A broad literature has already linked myosteatosis (muscle fatty infiltration), fibrosis, and atrophy to numerous spine pathologies, including hyper-kyphotic deformity^14–16^, intervertebral disc (IVD) degeneration^17,18^, IVD herniation^7,18^, and nonspecific low back pain^5^. Further, patients with kyphotic deformity of the lumbar spine present with greater paraspinal muscle degeneration compared to patients without deformity^6^. These spinal deformity-related muscle alterations are important, as the sagittal imbalance associated with kyphotic deformity has been shown to be the most reliable radiographic predictor and indicator of the health status of adults with spinal disorders^19^. While paraspinal and spinopelvic muscular dysfunction are hypothesized to be causative factors in degenerative spinal deformity^14,15,20–23^, experimental studies demonstrating true cause and effect relationships are lacking.

Recent work demonstrated large variability in the active (specific force) and passive (elastic modulus) functional properties of paraspinal muscle biopsies from degenerative adult spinal deformity patients, reporting values that appear to be impaired compared to age-matched literature skeletal muscle norms^24^. Nonetheless, whether muscle alterations are consequences of spine degeneration and deformity or whether they precede, drive, or accompany the disorder progression is unknown; causative data are needed to address such hypotheses. However, because of the long progressive time‐course of degenerative spine disorders in humans, and the lack of muscle data at asymptomatic early disease stages, it is unrealistic to obtain such data in humans. Few animal studies have attempted to untangle the relationship between paraspinal muscle pathophysiology and degenerative spinal deformities^25,26^. Cho et al.^25^ found that severe paraspinal muscle injury (2-week ischemia) in rats led to a thoracolumbar kyphotic deformity; however, the authors did not quantify the degenerative changes in the muscles and the mechanism of muscle injury was likely non-physiologic. Similarly, Hey et al.^26^ used a whole body TSC1 knockout myopathy model in mice, and demonstrated that whole body muscle myopathy leads to thoracolumbar kyphotic deformity in 12-month old mice when compared to age-matched controls. However, this study was limited in that the myopathy was not specific to the paraspinal muscles, but rather affected the entire skeletal muscle system, and the quantity of muscle fibrosis and fatty infiltration were not measured, nor were any functional measures performed.

Also of interest, is whether the active process of paraspinal muscle degeneration can initiate other spinal degenerative changes such as IVD degeneration. The opposite relationship, where the initiation of IVD degeneration leads to degenerative changes to the paraspinal muscles, has been demonstrated in multiple animal models^9,27–29^. Further study of the interrelationships that drive the initiation and progression of spinal degeneration, including paraspinal muscle and IVD degeneration and kyphotic deformity, is needed.

To better unravel the cause-and-effect association between paraspinal muscle degeneration and spine degeneration and deformity, we developed and characterized a novel model of paraspinal muscle degeneration using repeated experimental intramuscular glycerol injections in C57BL/6 wild type mice. It was hypothesized that the induced paraspinal muscle degeneration would lead directly to kyphotic spinal deformity and mild degeneration of the IVDs.

## Results

### Intramuscular glycerol injections induce paraspinal muscle degeneration

**Figure 1:**
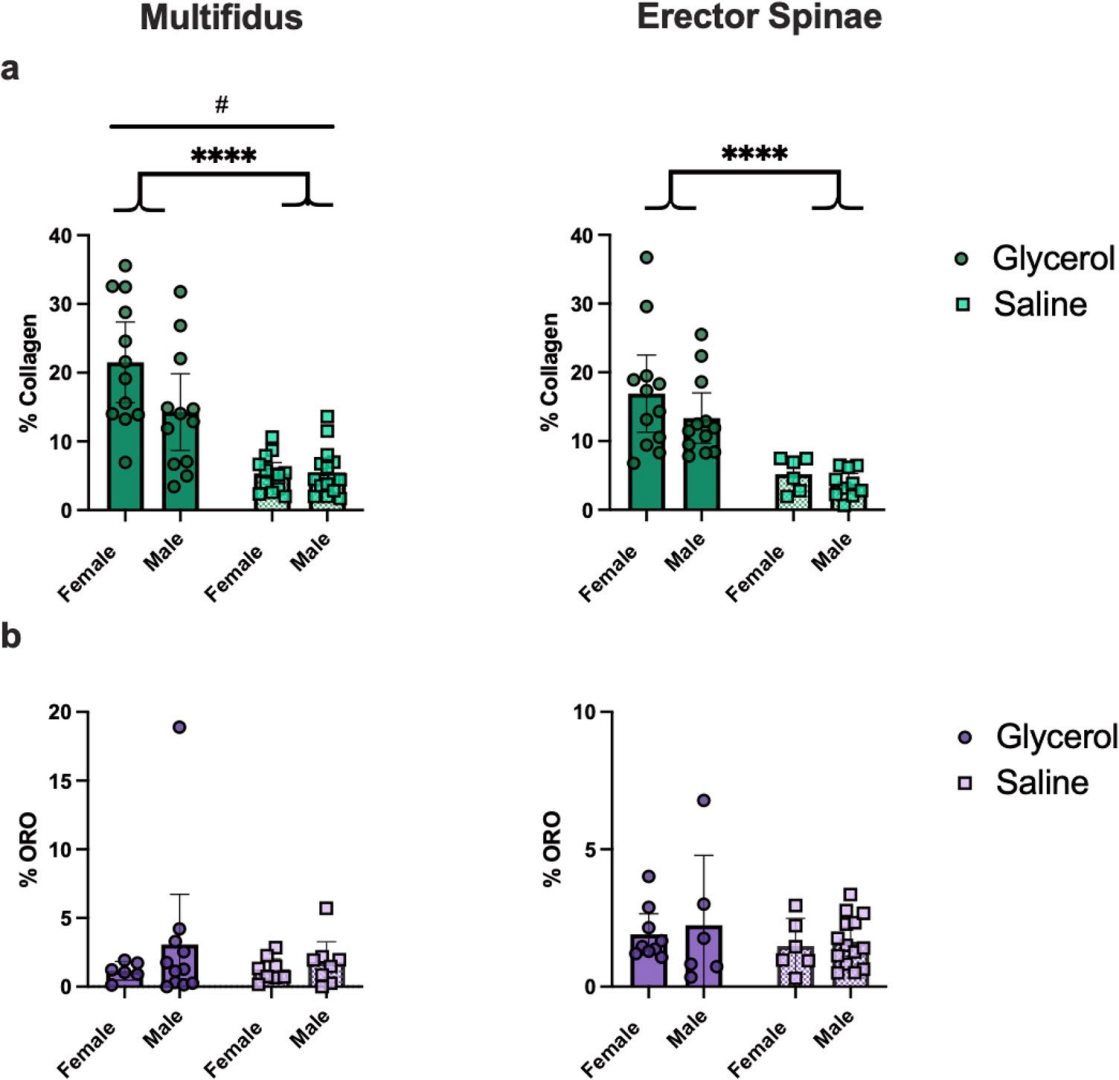
Repeated intramuscular glycerol injections induce fibrosis, but not fatty infiltration 14 days post final treatment. (**a**, **b**) Quantification of collagen deposition via picrosirius red + fast green staining indicates greater collagen content with glycerol treatment (squares: saline, circles: glycerol). (**c**, **d**) Quantification of fatty infiltration via Oil Red O (ORO) staining indicates no significant effect with glycerol treatment. N = 6 per group. (A) Main effect of group ****p < 0.01; #*p* = 0.047 represents a significant interaction effect between treatment group and sex. All data are reported as means ± 95% CI.

**Figure 2:**
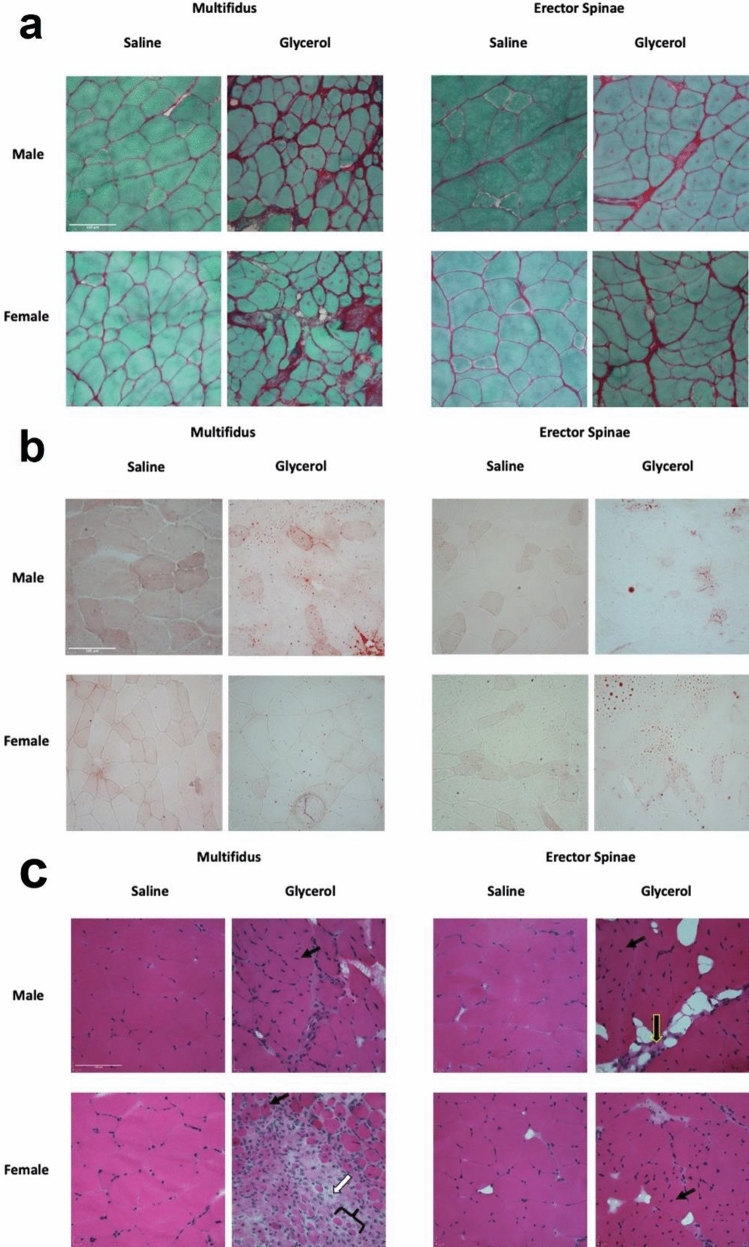
Representative images of male and female mice from both the glycerol and saline groups. (**a**) Picrosirius red + fast green, (**b**) ORO, (**c**) H&E. n = 1–2 sections per muscle. For (**c**) black solid arrow represents central nuclei indicative of regeneration (only some are highlighted); white solid arrow represents a mononucleated inflammatory cell; black solid brace represents small rounded fibres indicating de-/regeneration; black arrow with yellow outline represents muscle replacement by fibrotic tissue. Note that arrows and braces are representative, and that these features are widely apparent throughout the section. Scale bars = 100 µm.

As expected, glycerol injected mice demonstrated greater collagen content (i.e. fibrosis) compared to saline injected mice for both muscles (MULT: glycerol = 17.9% vs saline = 5.4%; main effect of group = *p* < 0.01; ES: glycerol = 15.1% vs saline = 4.5%; main effect of group = *p* < 0.0001) (Figs. 1, 2a). There was also an interaction effect in the MULT (*p* = 0.047), where the difference between glycerol and saline groups was greater in females than males. There was no main effect of sex (*p* = 0.056). In the ES muscle there was no interaction (*p* = 0.58) or sex effect (*p* = 0.22). Unexpectedly, glycerol injected mice did not demonstrate a significant difference in fat deposition compared to saline injected mice in either muscle (MULT: glycerol = 2.1% saline = 1.5%; main effect of group: *p* = 0.6296; ES: glycerol = 2.1% saline = 1.6%, main effect of group: *p* = 0.2797) (Figs. 1, 2b). Similarly, there were no effect of sex (MULT: *p* = 0.31; ES: *p* = 0.59) or interaction effects (MULT: *p* = 0.56; ES *p* = 0.85). Qualitatively, a greater number of central nuclei, more inhomogeneous fibre size distribution, and an infiltration of mononucleated inflammatory cells were found in the glycerol compared to saline groups (observed via H&E staining; Fig. 2c). Overall, these data indicate that multiple glycerol injections induce degeneration in the paraspinal muscles, characterized mainly by significantly greater collagen deposition (i.e. fibrosis), but not fat content.

### No change in CSA, but lower dorsal muscle density in mice injected with glycerol

**Figure 3:**
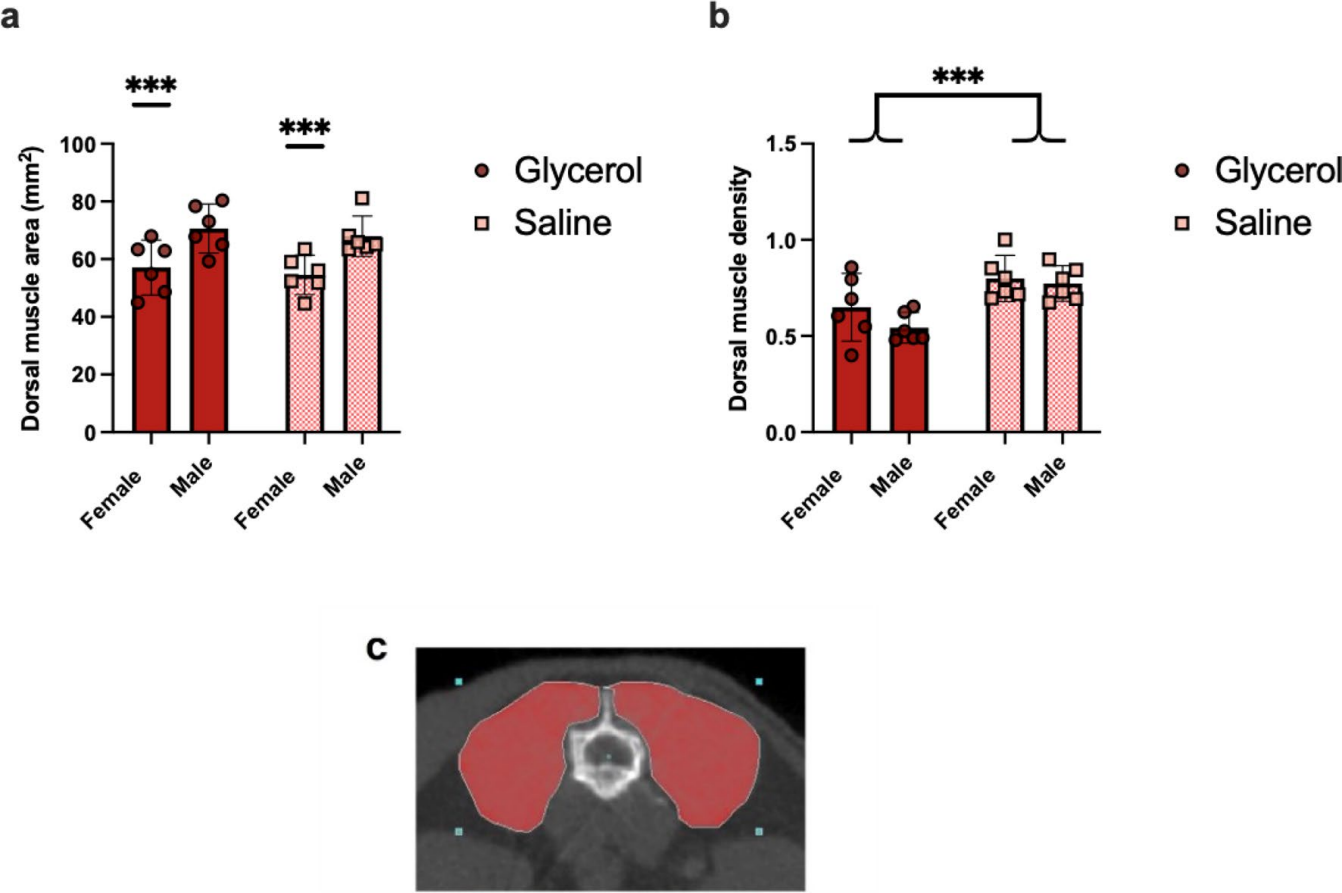
No difference in dorsal muscle CSA, but lower dorsal muscle density, in glycerol compared to saline treated mice measured 14 days following the final treatment. (**a**) Quantification of dorsal (multifidus, erector spinae and quadratus lumborum combined) muscle CSA demonstrates no group difference between glycerol and saline mice (*p* = 0.41). There was a significant difference between male and female mice (*p* < 0.01) with males have significantly larger dorsal muscle (represented by ***) CSA. (**b**) Quantification of dorsal muscle density indicates lower muscle density in glycerol-treated mice (*p* < 0.01). Note that the muscle density values are unitless as they were normalized to spleen density. N = 6 per group; light/squares = saline, dark/circles = glycerol. (**c**) Representative image of the dorsal muscle area region of interest (ROI) at L3 acquired with micro‐CT. All data are presented as means ± 95% CI.

There was no significant difference in the CSA (cross-sectional area) at the L3 level between glycerol and saline treated mice (Glycerol = 63.9 mm^2^ vs Saline = 61.3 mm^2^, *p* = 0.41) (Fig. 3a). There was a main effect of sex (males = 69.3 mm^2^ vs females = 55.8 mm^2^ *p* < 0.01) but no interaction (*p* = 0.98). For dorsal muscle density, a main effect of group was present (glycerol = 0.60 vs saline = 0.79 *p* < 0.01) but no effect of sex (male = 0.66 vs female = 0.72, *p* = 0.18) and no interaction (*p* = 0.41) (Fig. 3b).

### Isolated single fibres exhibit impaired absolute, but not specific force in the paraspinal muscles of glycerol treated animals 14 days post treatment

A total of 144 single muscle fibres were actively tested; from these, 128 were type IIB, 8 were type IIX, 1 was type IIA, 5 were type IIB/X, and 2 were type IIX/A. Therefore, only type IIB fibres were statistically tested and reported here.

**Figure 4:**
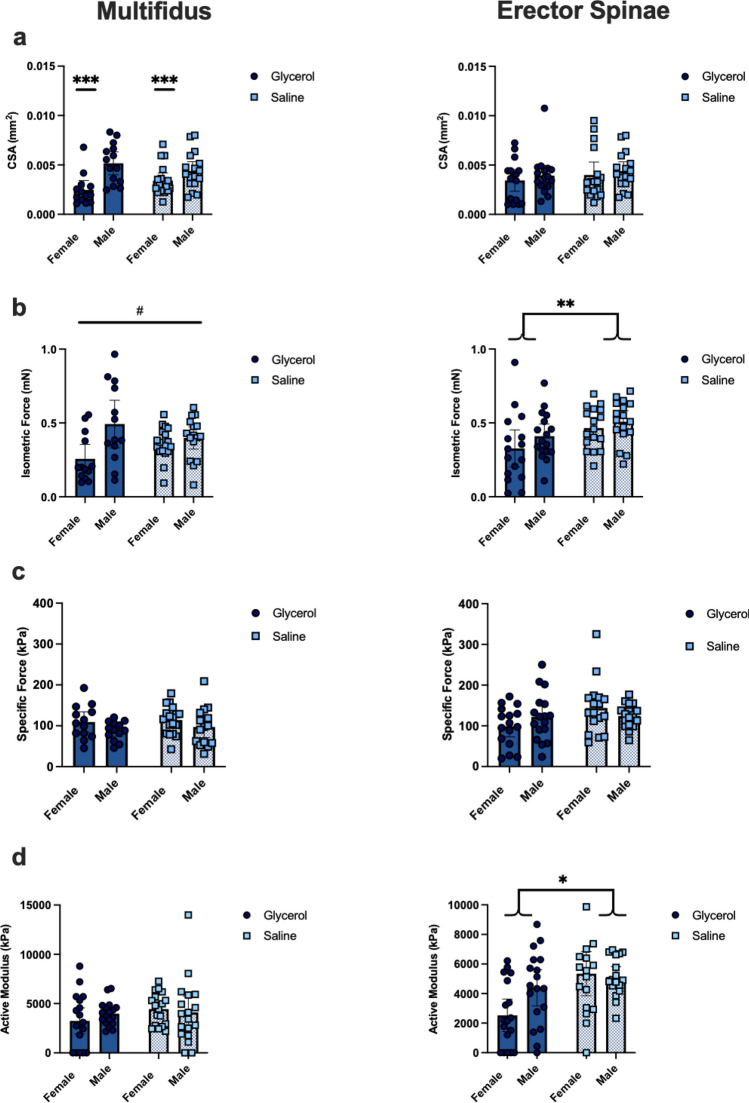
Repeated glycerol treatment results in lower absolute isometric force production of the paraspinal muscles. (**a**–**d**) Contractile measurements for MULT (left), ES (right) muscle fibres. (**a**) CSA, (**b**) steady-state absolute isometric force, (**c**) specific force, (**d**) active modulus. Each square (saline) or circle (glycerol) (MULT *n* = 59–65 per measure, ES *n* = 63–67 per measure) represents a single fibre. Values are presented as means ± 95% CI. *p = 0.01, significant effect of group; **p < 0.01, significant effect of group; ***p < 0.01, significant effect of sex; #*p* = 0.03 represents a significant interaction.

For single fibre CSA, there was no effect of group (MULT: *p* = 0.85; ES: *p* = 0.34) and no interaction, (MULT: *p* = 0.058; ES: *p* = 0.92) but a main effect of sex for the MULT (males larger CSA than females *p* < 0.01; ES: *p* = 0.41) (Fig. 4a). Peak steady-state absolute isometric force was lower in glycerol injected muscle compared to saline injected muscle in the ES (main effect *p* < 0.01) and the MULT in female but not male mice (group by sex interaction, *p* = 0.03; main effect of group, *p* = 0.91). In the ES there was no sex (*p* = 0.14) or interaction effect (*pp* = 0.63), and in the MULT there was a main effect of sex (*p* < 0.01) (Fig. 4b). Specific force was not different between groups (MULT: *p* = 0.82; ES: *p* = 0.07) nor was there an effect of sex (MULT: *p* = 0.06; ES: *p* = 0.90) or an interaction (MULT: *p* = 0.83 ES: *p* = 0.10) (Fig. 4c). Active modulus was not significantly different between groups (*p* = 0.57) nor was there an effect of sex (*p* = 0.74) or an interaction (*p* = 0.52) in the MULT; however, in the ES there was a significant effect of group (*p* = 0.01), where the glycerol group had a lower active modulus (i.e. lower active normalized stiffness) compared to the saline group, but no effect of sex (*p* = 0.587) and no interaction (*p* = 0.11) (Fig. 4d).

### Male mice have a faster rate of force redevelopment in single permeabilized fibres: no difference between glycerol and saline groups

**Figure 5:**
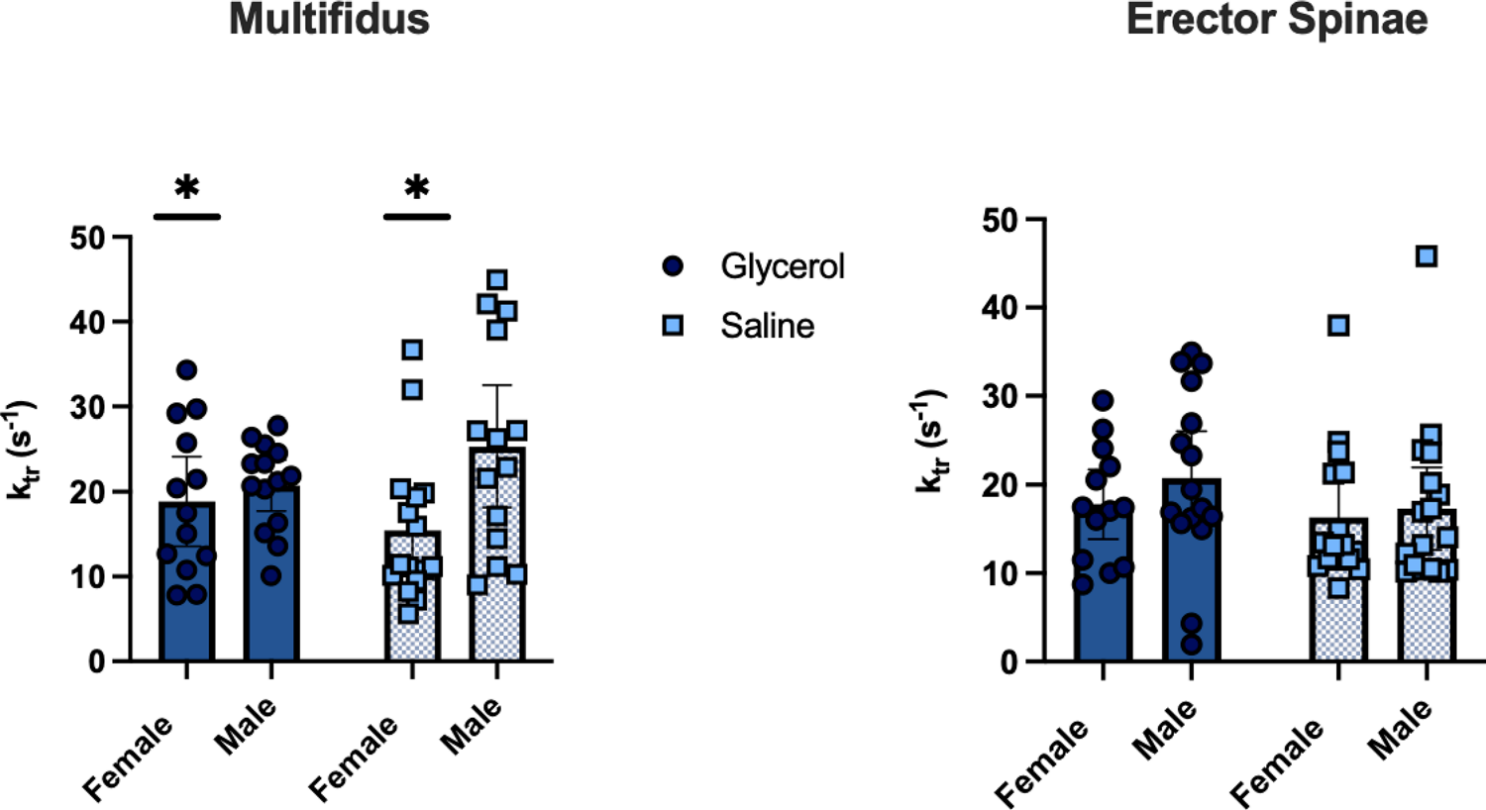
Rate of force redevelopment is not different in glycerol compared to saline treated mice. MULT (left) and ES (right) muscle fibres (MULT *n* = 57, ES *n* = 62). Values are presented as means ± 95% CI. *p = 0.02, significant effect of sex.

For rate of force redevelopment, there was no effect of group (MULT: *p* = 0.81; ES: *p* = 0.26) and no interaction effect (MULT: *p* = 0.11 ES: *p* = 0.66) in either muscle. However, there was a main effect of sex in the MULT (*p* = 0.02) with males having a 34% faster rate of force development than females. There were no sex differences in the ES (*p* = 0.36) (Fig. 5).

### Greater ES passive stiffness in glycerol injected mice

**Figure 6:**
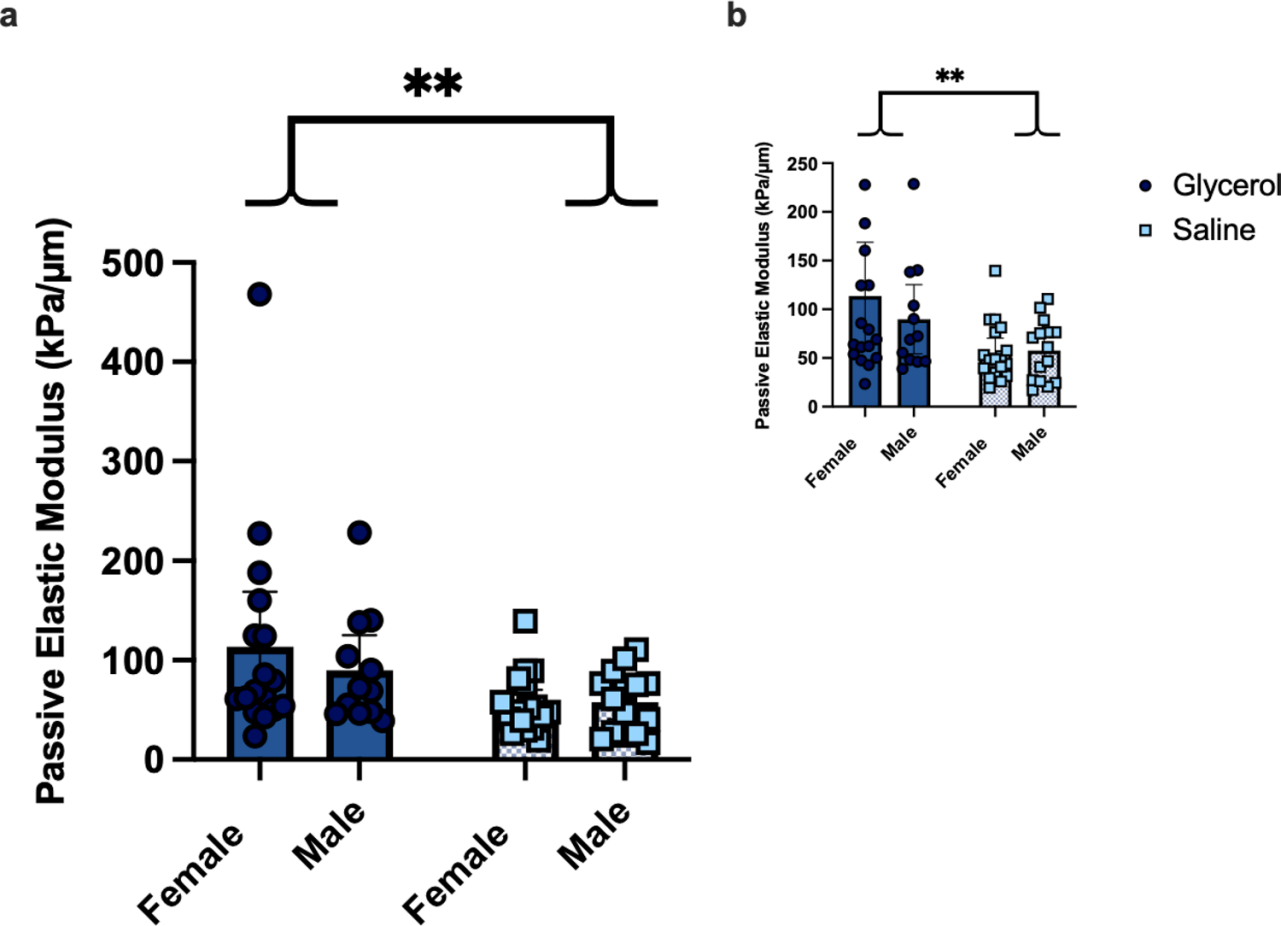
Glycerol treatment results in greater passive stiffness of muscle fibre bundles from the ES. (**a**) Passive mechanical measures for muscle fibre bundles from the ES (light squares = saline, dark circles = glycerol; n = 62 total fibre bundles). (**b**) Inset of figure A with outlier removed to better view the distribution of the data. Values are presented as means ± 95% CI. **p = 0.0092, significant effect of group (with or without outlier removed: with outlier removed *p* = 0.0045).

There was a significant effect of group (*p* < 0.01), with an 80% greater passive elastic modulus in the ES from the glycerol compared to saline injected groups. There was no effect of sex (*p* = 0.52) and no interaction effect (*p* = 0.44) (Fig. 6).

### Glycerol injected mice exhibit increased kyphotic deformity 2-weeks following the final injection: no changes in lumbar lordosis

**Figure 7:**
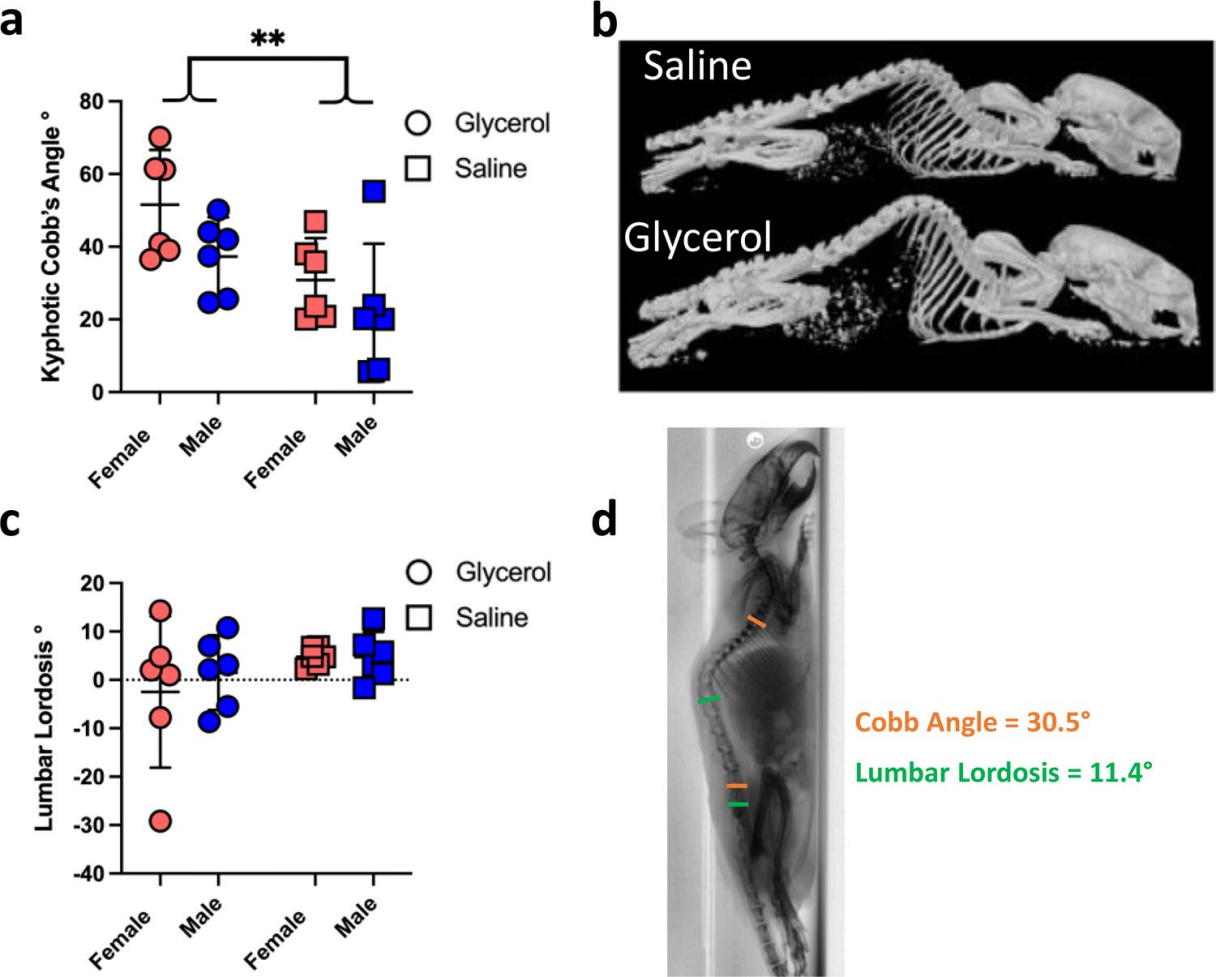
Paraspinal muscle myopathy leads directly to thoracolumbar kyphotic deformity but has no effect on lordosis. (**a**) Quantification of kyphotic deformity via Cobb Angle measurement demonstrating greater kyphosis in the glycerol compared to saline mice 14 days following the final injections. N = 6 per group; squares = saline: circles = glycerol. **p < 0.01. (**b**) 3-D model of representative female mouse skeletons, demonstrating greater thoracolumbar kyphosis in the glycerol mouse. top: saline; bottom: glycerol. (**c**) Lumbar lordosis angle in the glycerol compared to saline mice 14 days following the final injections. N = 6 per group; squares = saline: circles = glycerol. Note that the large confidence interval in the female glycerol mice is due to a single female with a large kyphotic (negative) lumbar angle. (**d**) Sagittal spinal alignment measurement using the Cobb method and lumbar lordosis with Surgimap software. All data are presented as means ± 95% CI.

Glycerol injected mice developed greater thoracolumbar kyphosis in comparison to saline injected mice. Specifically, glycerol injected mice demonstrated a greater kyphotic Cobb angle 2 weeks following the final injection (day 56) compared to saline injected mice (Glycerol = 44.4° vs Saline = 26.4° *p* < 0.01). There was no effect of sex (*p* = 0.052) and no interaction effect (*p* = 0.65) (Fig. 7a, b). Lumbar lordosis was not different between glycerol and saline injected animals (Glycerol = − 0.5° vs Saline = 4.8° *p* = 0.1552) with no effect of sex (*p* = 0.5915) and no interaction effect (*p* = 0.5758) (Fig. 7c).

### Glycerol injected mice demonstrate signs of mild IVD degeneration at the upper-most lumbar level

**Figure 8:**
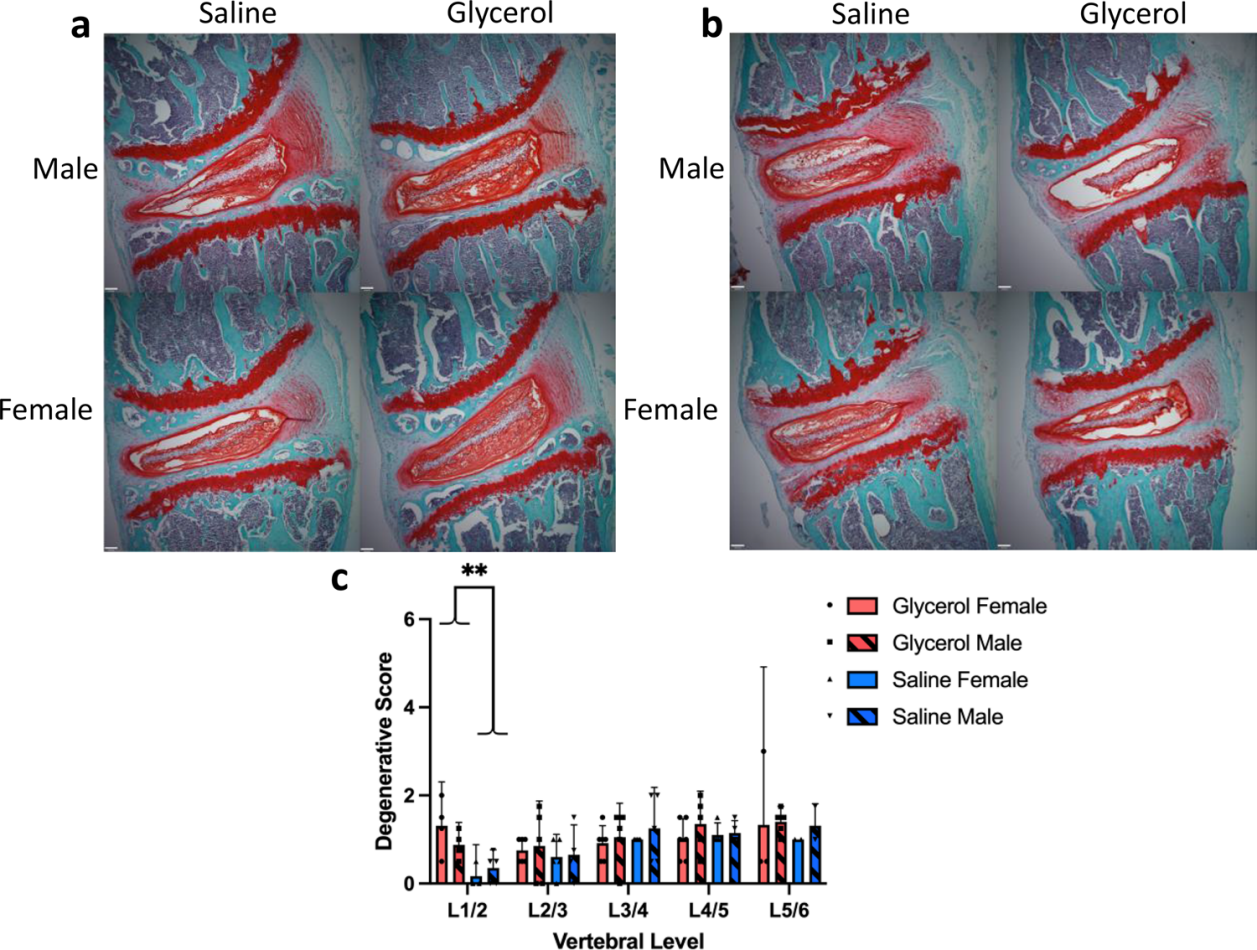
Effect of glycerol injections on IVD degeneration. Representative histological sections of (**a**) the L5/6 and (**b**) the L1/2 IVDs stained with safranin-o/fast green from male and female mice injected with glycerol or saline. Scale bars = 100 µm. (**c**) IVD degeneration scores (scale from 0 to 10) showed no significant difference between glycerol and saline injected mice at all levels except the L1/2 level. N = 6 animals/group. All data are presented as means ± 95% CI (with bars above only). **p = 0.0020. For each sex, 3–6 IVDs (individual data points) were analyzed per level, for each group.

There was no significant group difference in the degenerative score between glycerol and saline injected mice with the exception of the L1/2 vertebral level (Fig. 8): (L1/2 level: Glycerol = 1.1 Saline = 0.3, *p* = 0.002; L2/3 level: Glycerol = 0.8 Saline = 0.6, *p* = 0.47; L3/4 level: Glycerol = 1.0 Saline = 1.1, *p* = 0.5381; L4/5 level: Glycerol = 1.2 Saline = 1.1, *p* = 0.7837; L5/6 level: Glycerol = 1.4 Saline = 1.2, *p* = 0.6031). There was no significant effect of sex and no interaction effect for any level (L1/2 level: sex *p* = 0.5612, interaction *p* = 0.1701; L2/3 level: sex *p* = 0.7553, interaction *p* = 0.9172; L3/4 level: sex *p* = 0.4071, interaction *p* = 0.7989; L4/5 level: sex *p* = 0.2801, interaction *p* = 0.4143; L5/6 level: sex *p* = 0.6390, interaction *p* = 0.7603).

At the L1/2 level, the significant difference between the glycerol and saline groups was driven mainly by mild degenerative features noted in the nucleus pulposus (glycerol mean score = 0.59, saline mean score = 0.16), followed by the annulus fibrosus (glycerol = 0.38, saline = 0.06) and finally the nucleus/annulus boundary (glycerol = 0.13, saline = 0.06).

## Discussion

The pathophysiology driving degenerative spinal disorders and spinal deformity is complex and has been suggested to involve the paraspinal muscles^14,15,20–23^. However, direct evidence in support of this hypothesis is incomplete. Here, employing a mouse model of repeated intramuscular glycerol injections over an 8-week period, it is shown that the paraspinal muscles of glycerol injected mice have significantly more fibrotic tissue, greater number of central nuclei (inferring that the muscle is undergoing cycles of degeneration and regeneration), produce less absolute active force, and are passively stiffer. Further, a direct link is established between these unfavourable muscle properties and the development of thoracolumbar hyper-kyphotic deformity. The main findings of this study are that (1) paraspinal degeneration can lead directly to hyper-kyphotic deformity; the kyphotic Cobb’s angle was significantly (68%) greater in the glycerol compared to the saline injected mice at the final timepoint (day 56); (2) paraspinal muscle weakness (glycerol injected muscles had ES muscle fibres that were 24% weaker than saline injected muscles) and passive stiffness (glycerol injected ES muscles had fibre bundles that were 80% stiffer than saline injected ES muscles) appear to be the dominant muscle functional parameters leading to the hyper-kyphotic deformity in this model; (3) the hyper-kyphotic deformity is not accompanied by significant IVD degeneration, but there are signs of mild IVD degeneration developing at the upper-most lumbar level. These findings provide direct evidence that both morphological (fibrosis) and functional (actively weaker and passively stiffer) alterations to the paraspinal muscle compartment can drive negative changes to the thoracolumbar spine—evidence that has been lacking in the literature to date.

Limited studies have attempted to demonstrate that paraspinal muscle pathophysiology can precede, and directly lead to, negative changes to the spine (e.g.^25^). Previous studies have proposed that kyphotic deformity develops because of postural changes—as a method of conserving energy^26^, while others have suggested that intervertebral disc degeneration, which can lead to a loss of disc height and bone remodelling, could contribute to the onset of deformity^15,30,31^. Finally, many have hypothesized that paraspinal and spinopelvic muscular dysfunction are causative factors in the development of spinal deformity^14,15,20–23^. While presenting compelling hypotheses, these studies are unable to unravel the sequence of events that lead to kyphotic deformity. This gap in the literature is significant as the sagittal imbalance that arises alongside kyphotic deformity has been shown to be the most reliable radiographic predictor and indicator of the health status of adults with spinal disorders^19^.

The paraspinal muscles of patients with LBPDs are prone to developing myostaeosis (intrusion of fatty tissue into the body of the muscle), and fibrosis (tissue remodeling whereby muscle tissue is replaced by collagen-based connective tissue). For example, fatty and/or fibrotic changes have been regularly observed using non-invasive imaging in IVD herniation^5,32^, nonspecific low back pain^4,33,34^, and spinal stenosis^35^, including greater fatty/fibrotic infiltration in stenosis patients who have a lower compared to higher functional status^35,36^. Fatty-fibrotic changes have also been observed histologically using muscle biopsies taken from patients during surgery to treat IVD herniation^6,7,37^. Further, it appears that patients with kyphotic deformity of the lumbar spine display greater degeneration of their paraspinal muscles compared to patients without deformity^6^. For example, Delisle et al.^6^ found that the paraspinal muscles from patients with progressive lumbar kyphosis had more extensive fibrosis than in patients being treated for IVD herniations. Further, Malakoutian et al.^24^, using histological analyses of intraoperative biopsies, revealed that adult spinal deformity patients had frequent fibro-fatty replacement, which lead to higher passive stiffness values than those reported in the literature from non-deformity patients, and identified a range of muscle fibre abnormalities in the biopsies. Finally, paraspinal muscle weakness and dysfunction are hypothesized to be causative factors in degenerative spinal deformity^14,15,20–23^; however experimental confirmation of this hypothesis has been lacking.

The intramuscular glycerol injection model employed here induced a significant amount of fibrosis, and a subsequent increase in passive muscle stiffness in the paraspinal muscles, which mimics the findings of histological studies demonstrating greater amounts of fibrotic tissue in chronic lumbar spine pathology^37^, kyphotic deformity^6^, combined kyphotic and scoliotic deformity^24^, and IVD herniation^7^ patients. However, the current glycerol model did not induce a significant amount of fatty infiltration as quantified by ORO staining, which is largely in contrast to previous studies using single glycerol injections into mouse limb muscles^38^ and reporting on human LBPD patients where fatty infiltration is generally prominent within the paraspinal muscles using non-invasive imaging (e.g.,^5,32^).

While there is a growing body of human literature demonstrating links between altered paraspinal muscle morphology and LBPDs, much is still unknown regarding their direct mechanistic interaction. Specific to deformity, Cho et al.^25^ found that severe paraspinal muscle injury (2-week ischemia) in rats led to a thoracolumbar kyphotic deformity that persisted for the remainder of the study (12 weeks); however, the authors did not quantify the degenerative changes in the muscles. A recent study knocked-out the TSC1 gene in female mice to create a myopathy within the entire skeletal muscle system and revealed that whole body muscle myopathy leads to thoracolumbar kyphotic deformity in 12-month, but not 9-month-old mice when compared to age-matched controls^26^. This study^26^ was limited in that the myopathy was not specific to the paraspinal muscles, but rather affected the entire skeletal muscle system, and the quantity of fibrosis and fatty infiltration were not measured, nor were any functional measures performed. Other mouse models with known whole body skeletal muscle weakness (e.g. Mdx mice^39^ and tetranectin-deficient mice^40^) have also been shown to develop spinal hyper-kyphosis.

Recent work by Lorbergs et al.^41^ reported that a smaller cross-sectional area and lower quality of thoracic paraspinal muscles were associated with a larger kyphotic Cobb angle in a population aged 50 years and above. In the current study, micro-CT analysis provided broader measures of dorsal muscle CSA (cross-sectional area) and density (the latter a representation of muscle quality) at the L3 vertebral level. While glycerol injections did not alter the overall dorsal muscle CSA, dorsal muscle density was significantly lower in glycerol injected compared to saline injected mice. This lower density is reflective of a lower percentage of the muscle occupied by contractile tissue which again demonstrates a direct link to the development of spinal deformity.

To our knowledge this is the first study to investigate whether impaired paraspinal muscle function can precede and thus lead to spinal deformity. Here, lower absolute force production was apparent in the ES (both sexes) and MULT (female only) in the glycerol injected compared to saline injected group. As neither fibre CSA nor specific force were statistically different between glycerol and saline groups, it is likely the combination of small differences in both (i.e. slightly lower CSA and specific force in the glycerol group) that leads to the larger and statistically significant differences in absolute force. This is partially supported in the ES by the significant difference in active modulus (25% lower in the glycerol group), suggesting that even when accounting for the size of the fibre, there were fewer attached cross bridges during active steady-state isometric force production. This implies that ES specific force may have been trending towards being significantly lower in the glycerol group. Most notably, a clear and significant difference was observed in the passive elastic modulus between groups for the ES (80% greater modulus in the glycerol compared to the saline group; not measured in the MULT due to lack of tissue), likely a result of the significantly greater amount of collagen in the muscle. The sensitivity to passive muscle remodelling is not surprising, as previous studies have also revealed changes to the passive mechanical properties of the spine muscles in response to spine pathology^9,42^; however, this is the first study to show the inverse relationship, whereby changes to passive muscle properties likely precede pathological changes to the spine. Future studies should investigate multiple timepoints using in-vivo imaging and/or the addition of more animals to improve precision over the exact timing of events driving changes to the paraspinal muscles and the development of spinal deformity.

The results of the IVD analyses demonstrate, at most, the presence of mild degeneration (mean scores across all levels of 1.1/10 in the glycerol groups, and 0.9/10 in the saline groups). Statistically significant differences between glycerol and saline groups were present only at the L1/2 level (mean score of 1.1 in the glycerol group and 0.3 in the saline group). While this L1/2 score of 1.1 (out of 10) in the glycerol group represents very mild degeneration, L1/2 was the most cranial level analyzed, thus representing the level closest to the onset of the deformity. As such, the small difference between the glycerol and saline groups at this (L1/2) vertebral level may be indicative of accelerated degeneration in the glycerol group that would have progressed further over a longer time period and/or the presence of progressing degeneration in IVDs located more cranially in the region of deformity. Despite this possibility, it is clear that the development of deformity has preceded any meaningful IVD degeneration in the lumbar region.

Two muscle measures demonstrated a stronger effect in female compared to male mice. The first, collagen content as quantified by histology, was greater in the glycerol injected muscles of both males and females, but the effect was significantly larger in the MULT of females compared to males. The second, absolute force, was significantly lower in both sexes of the ES in glycerol compared to saline muscles, but in MULT was only lower in the glycerol compared to saline muscles of females and not males. Despite these stronger degenerative phenotypes in the female compared to male muscles, there was no sex-based difference in the magnitude of kyphotic deformity induced by glycerol injections.

It should be noted that while the current results demonstrate a clear link between paraspinal muscle myopathy and spinal deformity, this should not be directly extrapolated to humans, who have clear functional differences when compared to rodents (e.g. biped vs quadruped), and whose lumbar spine is lordotic (humans) while rodents have a much flatter lumbar region.

In summary, this study has successfully demonstrated that: (1) experimental intramuscular glycerol injections in C57BL/6 wild type mice lead to muscle degeneration including severe muscle fibrosis, passive muscle stiffening and impaired absolute force production. Interestingly, multiple glycerol injections in mice did not lead to a significant increase in fatty infiltration within the paraspinal muscles. (2) Paraspinal muscle myopathy leads directly to kyphotic spinal deformity; thus, providing the first direct evidence that paraspinal degeneration can initiate the development of spine deformity.

## Methods

### Animals

Experiments were performed on 10–12-week-old female (n = 12) and male (n = 12) C57BL/6 mice (Charles River Laboratories) approved by the University of Guelph Animal Utilization Protocol (#4533) in accordance with all relevant guidelines and regulations; reporting of methods here follow ARRIVE guidelines. All mice were 22–26 g at the start of the experiment and allowed free cage activity and ad libitum access to food and water. Mice were housed in standard 22 °C conditions. Twelve mice (6 female and 6 male) received bilateral glycerol injections to the mid-belly of the multifidus and erector spinae muscles along the ~ L1–L6 vertebral levels to induce muscle myopathy. Twelve control mice (6 female and 6 male) were injected identically, but with saline instead of glycerol. Glycerol provokes muscle regeneration by inducing myofibre necrosis and intramuscular fat deposition in mice^38^ and has been shown to induce early fibrosis in the rat.^43^ Here injections were performed every 14 days for 42 days (4 injection timepoints) with the aim of creating a degenerative environment within the paraspinal muscle compartment.

### Intramuscular injections

Mice were continuously anaesthetized with 2% inhaled isoflurane at 2 L/min and injected subcutaneously over the incision site with a 50/50 lidocaine-marcaine mixture. The depth of anaesthesia was assessed by toe pinch. The dorsal side of the mouse was shaved and sterilized with betadine. A 2–3 cm incision was made through the skin over the lumbar spine exposing the multifidus and erector spinae muscles. 15 μl of either glycerol (50% v/v) or sterile saline was then injected bilaterally in each of the lumbar multifidus and erector spinae, from L1 to L6, with a 29.5-gauge insulin syringe. Skin incisions were closed with EZ Clips. Mice were allowed free cage activity and were monitored daily for signs of pain, distress, and infection. Intramuscular injections were performed every 14 days for 42 days (i.e., 4 injection timepoints); mice were sacrificed 14 days following the final injection under anaesthesia followed by CO_2_ asphyxiation.

### Micro-computed tomography

Micro‐CT imaging was performed immediately following sacrifice to measure paraspinal muscle cross-sectional area (CSA), muscle density, and spinal deformity. Scanning was performed with a Skyscan 1278 (Bruker micro‐CT, Kontich, Belgium) at 50 μm voxel resolution using a source voltage of 48 kV and a current of 1030 μA. Aluminium filter was set at 0.5 mm to optimize contrast while minimizing dose. Rotation step for the X‐ray source was set to 0.7°. Average scanning time per animal was 2.5 min. Raw images were then reconstructed with NRecon to 3D cross‐sectional image data sets using the following parameters: beam hardening to 20%, smoothing to 2%, minimum and maximum for CS to Image Conversion to 0% and 0.03%, respectively. Analyses of muscle CSA and density from reconstructed images were performed using SkyScan software (CTan). To measure CSA (mm^2^) and density [in Hounsfield units (HU)], the multifidus, erector spinae, and quadratus lumborum were combined and will be referred to as ‘dorsal muscle’; muscles were combined because they couldn’t be separated reliably, similar to previous publications in the field.^44^ First, a grey value threshold (33–90) was applied to exclude non‐lean tissue (i.e. mostly bone), then a region of interest was manually drawn on the dorsal muscle area at L3 (Fig. 3c). Dorsal muscle area (mm^2^) was normalized to body mass, and dorsal muscle density was normalized to spleen density (an internal invariant control). The muscle‐to‐spleen ratio is referred to here as muscle density^44^.

Sagittal spinal alignment was measured by calculating both Cobb (measured from the inferior endplate of L5 to the superior endplate of T5) and lordosis (superior endplate of S1 to the superior endplate of L1) angles with Surgimap software (version 2.3.2.1) (Fig. 7d).

### Muscle biopsies and histology

Biopsies of multifidus and erector spinae from saline and glycerol injected mice were harvested immediately after the Micro-CT and split into two pieces each. The first were immediately placed into a cold dissection solution for contractile and mechanical testing (described later). The second pieces were embedded in OCT compound (Tissue-Tek), frozen in liquid nitrogen-cooled isopentane, stored at − 80 °C, and cut into 10-μm-thick cryosections with a cryostat (Leica CM1850) maintained at − 20 °C. Histological staining included hematoxylin and eosin (H&E), picrosirius red + fast green (PR + FG), and oil red O (ORO); PR + FG and ORO were used to detect collagen and fat, respectively. One to two images per muscle per stain were acquired with a brightfield Leica DM 5000B microscope connected to a Hamamatsu Orca-Flash 4.0 digital camera and Velocity imaging software. PR + FG and ORO images were thresholded in ImageJ and the red-positive area (both collagen and fat are stained red in the PR + FG and ORO stains, respectively) was divided by the total area to provide a measure of collagen and fat area fractions.

### Muscle permeabilization

The muscle pieces from the cold dissection solution were further dissected into fibre bundles 3–5 mm in length and ~ 0.5 mm in diameter. Following dissection, bundles were immersed for 30 min in skinning solution with 0.5% non-ionic detergent Brij 58 and then placed in storage solution and maintained for 24 h at 4 °C, followed by storage at − 80 °C.^45^ On the day of a contractile or passive mechanical experiment, fibre bundles were removed from storage solution and placed in relaxing solution on ice.

### Muscle solutions

The storage solution was composed of (in mM) 250 K-propionate, 40 Imidazole, 10 EGTA, 4 MgCl_2_·6H_2_O, and 2 ATP, and dissolved in glycerol so that the final volume of glycerol was 50% v/v. The skinning solution was identical to the storage solution except glycerol was replaced by deionized water and 0.5% w/v Brij 58 was added. Relaxing solution consisted of (in mM) 59.4 imidazole, 86 KMSA, 0.13 Ca(MSA)_2_, 10.8 Mg(MSA)_2_, 5.5 K_3_ EGTA, 1 KH_2_PO_4_, 0.05 leupeptin, and 5.1 Na_2_ATP. The pre-activating solution consisted of KPr (185), MOPS (20), Mg(CH_3_COOH)_2_ (2.5), ATP (2.5) while the activating solution contained Ca^2+^ (15.11), Mg (6.93), EGTA (15), MOPS (80), ATP (5), CP (15), K (43.27), Na (13.09), H_2_O. All solutions were adjusted to a pH of 7.0 with the appropriate acid (HCl) or base (Tris).

### Muscle contractile measurements

Briefly, single fibres were carefully removed from bundles and transferred to an experimental chamber containing relaxing solution maintained at 15 °C. There, fibres were tied with one end secured via monofilament nylon sutures to a pin in series with a force transducer (Aurora Scientific, model 403A) and the other end secured in a similar manner, to the lever arm of a servomotor (Aurora Scientific, model 322C). The length of the fibre was adjusted to obtain the target sarcomere length using a high-speed camera (HVSL, Aurora Scientific 901B). Fibre length (L_o_) was measured by aligning the innermost portion of the nylon tie at each end of the fibre with the crosshairs of a microscope eyepiece graticule. Measurements of fibre diameter were taken at 3 locations along the fibre length using a micromanipulator; from these measurements fibre CSA was calculated (assuming a cylindrical shape).

Relaxed single fibres were set slightly beyond their expected optimal length (~ 2.5 µm—to account for internal shortening) and activated by first immersing them in a chamber containing a pre-activating solution for 30 s and then moving them to a chamber containing a high-Ca^2+^ activating solution (pCa 4.2) to elicit maximal force. Force and length data were sampled at a rate of 10,000 Hz. Maximal force was calculated as the peak amplitude (peak force achieved in activating solution minus the resting force in relaxing solution), which was then divided by the CSA of the muscle fibre to give a measure of *specific force* (*Sf*_*o*_). Once maximal force was developed, *active modulus* (i.e., normalized instantaneous stiffness) was assessed by inducing a rapid (500 L_o_/s) stretch of 0.3% of L_o_ and dividing the change in force (normalized to CSA) during the stretch by the length change (normalized to L_o_).

Again, at maximal force, an additional length step was induced to measure *rate of force redevelopment (k*_*tr*_*).* This was done by rapidly shortening the fibre by 15% of L_o_ at a rate of 10 L_o_/s followed by a rapid (500 L_o_/s) re-stretch back to L_o_. The rapid shortening causes all cross-bridges to break, and then the re-stretch allows further dissociation of any remaining cross-bridges and redevelopment of force independent of Ca^2+^-dependent regulatory proteins at L_o_. A mono- exponential equation, y = a (1 − e^−kt^) + b, was fit to the redevelopment curve to determine *k*_*tr*_^46,47^. After completion of the contractile testing, fibres were placed in 15 μl of solubilization buffer and stored at − 80 °C for a minimum of 48 h. The myosin heavy chain (MHC) composition of each fibre (i.e. fibre type) was determined by sodium dodecyl sulfate–polyacrylamide gel electrophoresis (SDS–PAGE)^48^.

### Muscle passive measurements

For passive measurements, testing was performed in relaxing solution. Two to three muscle fibre bundles (8–20 single fibres ensheathed in their extracellular matrix) were dissected and tested from each erector spinae muscle sample. Bundles were then tied at either end to two separate pins: one attached to a force transducer (resolution 10 mN; Model-405A, Aurora Scientific, Inc., Aurora, ON, Canada), the other to a lever arm of a servomotor (Model-322C, Aurora Scientific, Inc.). Bundles were set to their slack length (length at which passive resistance to stretch was first detected) and measurements of diameter were taken at three locations along the bundle length using a digital micromanipulator (precision 1 µm) while viewed under a stereo microscope. Bundles were transilluminated at their approximate mid-length by a 5-mW diode laser (beam diameter ~ 0.5 mm; Coherent, Wilsonview, OR) and the resultant diffraction pattern was used to calculate sarcomere length^49^. Force and sarcomere length were recorded as bundles were rapidly stretched (at a rate of two bundle lengths per second) by cumulative increments of ~ 0.25 µm/sarcomere. For example, if slack sarcomere length was 2.1 µm, the first stretch would reach a mean sarcomere length of approximately 2.35 µm, the second stretch would occur from a mean sarcomere length of approximately 2.35–2.60 µm, and so on; for each test, 5–7 stretches were required for the test to be successful. After each stretch, sarcomere length was measured and the bundle was allowed to relax for 2 min before force was recorded and the next stretch was performed. This force was normalized to the CSA calculated from the average of the diameter measures (assuming a cylindrical shape) to give a value of stress. For each bundle a, passive stress-sarcomere length curve was generated, and the slope of the linear portion of this curve (beyond approximately 3.4 µm) was calculated to determine the passive elastic modulus.

### Intervertebral disc histology

Lumbar spines (intact L1-L6 vertebral levels) were harvested from both saline and glycerol injected C57BL/6 mice. Spines were fixed and decalcified for 3 days in Cal-Ex™ II (Fisher Scientific™). After decalcification, the spines were transferred to a cassette and placed into a 70% ethanol solution before being dehydrated in isopropanol, cleared in xylene, and infiltrated with paraffin. Sagittal plane sections (5 µm) were made through the approximate spine midline and stained using Safranin-O Fast Green. Sections were cover slipped and imaged at 20X on a brightfield Leica DM 5000B microscope connected to a Hamamatsu Orca-Flash 4.0 digital camera and Velocity imaging software, followed by scoring by two blinded evaluators using a modified scoring criteria^50^, where the nucleus pulposus, annulus fibrosus, and the nucleus/annulus boundary were graded from 0 to 4, 0 to 4, and 0 to 2, respectively, and the total scores summed (for minimum and maximum scores of 0 and 10). Data are presented as the scores averaged from both evaluators.

### Statistics

All data were analysed by two-way analysis of variance (ANOVA), with factors of group (glycerol and saline) and sex (male and female). If a significant interaction was discovered, Sidak multiple comparisons were used to compare individual group differences between males and females within the two-way ANOVA. Significance was set to α = 0.05. All data are reported as means ± 95% CI.

## Data Availability

Data available upon request to the corresponding author (S.H.M. Brown).
